# Factors Associated with Vaccination Status of Neonates in the Tertiary Referral Department of Neonatology and Neonatal Intensive Care in the North-Eastern Region of Poland

**DOI:** 10.3390/vaccines13121191

**Published:** 2025-11-25

**Authors:** Aleksander Kamianowski, Cezary Kamianowski, Gabriela Szpica, Angelika Jakubas, Anna Wasilewska, Monika Kamianowska

**Affiliations:** 1 Department of Neonatology and Neonatal Intensive Care, Medical University of Bialystok, 15-174 Białystok, Poland; 2 Department of Pediatrics and Nephrology, Medical University of Bialystok, 15-274 Białystok, Poland

**Keywords:** vaccination, neonatology, BCG vaccine, hepatitis B vaccine, vaccine hesitancy, parental refusal

## Abstract

**Introduction**: Effective strategies to increase vaccination acceptance should be targeted to a given community. We decided to conduct a study analyzing the immunization status of neonates and factors influencing it in the Department of Neonatology and Neonatal Intensive Care of the Medical University of Bialystok, Poland. **Material and Methods**: The retrospective study was conducted between 2015 and 2024. Vaccinations against tuberculosis (Bacillus Calmette-Guérin (BCG) vaccine) and against hepatitis B (1st dose) were analyzed. The multivariate logistic regression was used to assess the relationship between immunization status and neonates’ characteristic. **Results**: 88.35% of the neonates (N = 18,643) received both vaccines. Of the 2459 unvaccinated neonates, 965 (39.24%) were due to parental refusal, with 720 (74.61% of this subgroup) refusing both vaccines. The fact that the neonate did not receive both vaccines was associated with the following variables: mother’s age (*p* = 0.004), place of residence (*p* = 0.012), parity (*p* = 0.002), and gestational age (*p* = 0.000). **Conclusions**: The analysis revealed a specific group of neonates who are at risk of not receiving both vaccines: term neonates born to multiparous mothers aged ≥35 years and living in cities. These results may suggest which patients, in particular, should be taken into account when designing strategies to increase vaccine acceptance in the area covered by the study.

## 1. Introduction

Vaccination is considered one of the most successful public health achievements as it has significantly reduced both morbidity and mortality from various infectious diseases [1]. However, concerns about safety, effectiveness, and necessity of vaccination have been exacerbated in recent years, especially after the COVID-19 (coronavirus disease 2019) pandemic. The pediatric population is especially affected by this phenomenon and concerns regarding this issue are even more visible in its most vulnerable part—neonates [2]. In recent years, anti-vaccination campaigns have been increasingly observed in Central and Eastern Europe as well as in Poland [3,4]. Their influence on parents’ decisions regarding vaccination of neonates is clearly noticeable [5,6]. Parental concerns lead to varying decisions, including complete refusal of all vaccinations. As a result, lack of confidence in vaccines and vaccine hesitancy is nowadays considered a threat to public health [7,8].

Individual decision-making regarding the vaccination of a child is a complex process involving cultural, social, emotional, spiritual, political, and cognitive factors which are closely related to the region in which a given person lives [8,9,10]. Nowadays, growing concerns about vaccines have led to the development of various strategies to increase vaccine acceptance. However, these strategies must be targeted to a given community’s characteristics and requirements. For this reason, vaccination coverage analyses should be carried out in each part of the world [3,8].

In light of the above-mentioned factors, we decided to conduct a study analyzing the immunization status of neonates and factors that may influence it. The study was carried out in the Department of Neonatology and Neonatal Intensive Care of the Medical University of Bialystok, Poland. Vaccinations performed before discharge from the Department (against tuberculosis and against hepatitis B (1st dose)) were analyzed.

## 2. Material and Methods

This descriptive and analytical retrospective study was conducted in the Department of Neonatology and Neonatal Intensive Care of the Medical University of Bialystok between 1 January 2015 and 31 December 2024. The center where the study was conducted is the main tertiary referral hospital in north-eastern Poland and is representative of the medical facilities offering neonatal care in this area.

### 2.1. Recommendations on Vaccination of Neonates in Poland

In Poland, according to the vaccination calendar, a neonate should be vaccinated against (1) hepatitis B (1st dose) within 24 h after birth and (2) tuberculosis (Bacillus Calmette-Guérin (BCG) vaccine) within 24 h after birth or later, before discharge from the hospital [11].

### 2.2. Subjects and Data Collection

All neonates consecutively admitted to the Department during the study period were included in the study. The only reason for the exclusion was missing data in medical record. Particular attention was paid to checking the records for vaccination status. If complete data on the characteristics and vaccination of a neonate could not be found, it was excluded from further analysis.

We collected data using a structured questionnaire, which included the following variables: sex, mode of delivery, type of pregnancy (single and multiple), maternal age, gravidity and parity, place of residence, birth weight, body weight, gestational age, and 1 min Apgar score. Data entered into the database was encoded to ensure patient privacy.

**Figure 1:**
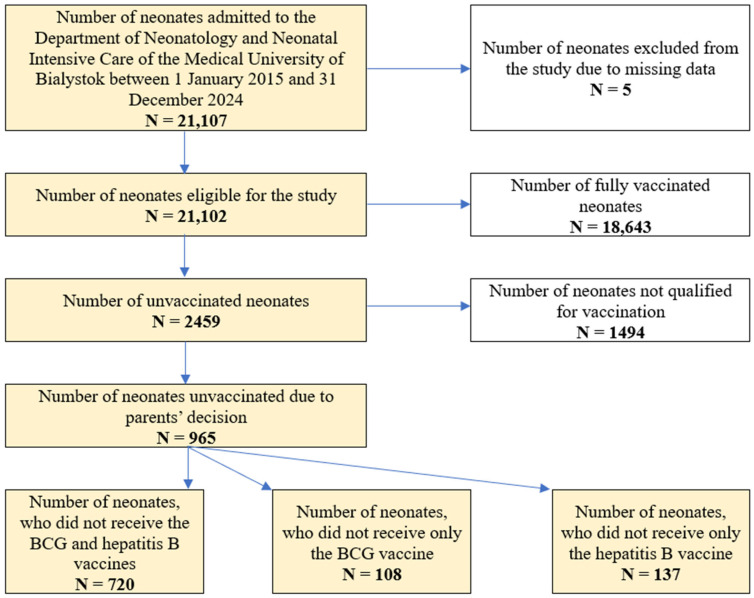
Flow diagram of the study group.

The neonates were categorized into 3 main groups: A—fully vaccinated neonates, B—neonates not fully vaccinated due to parents’ decision, and C—neonates not qualified for vaccination. The group B was then divided into 3 subgroups: B1—neonates, who did not receive the BCG and hepatitis B vaccines, B2—neonates, who did not receive the BCG vaccine (but received the hepatitis B vaccine), B3—neonates, who did not receive the hepatitis B vaccine (but received the BCG vaccine). The flow diagram of the study group is presented in Figure 1.

### 2.3. Study Covariates

To assess the determinants of immunization status of the neonates, numerous neonatal, maternal, and household-related covariates were examined. Among the neonatal factors, sex, gestational age, 1 min Apgar score, birth weight, and body weight were assessed. The following maternal factors were included: type of pregnancy, mode of delivery, maternal age, gravidity, and parity. Place of residence was assessed as a household factor.

### 2.4. Assessment of the Neonatal Immunization Before, During and After the COVID-19 Pandemic

The assessment of changes in the percentage of vaccinated and unvaccinated children was also performed separately for the following time periods: before, during, and after the COVID-19 pandemic. The periods were determined by taking into account the beginning—11 March 2020—and the end of the COVID 19 pandemic—5 May 2023 [12]. Finally, the following periods were distinguished: (1) before the COVID-19 pandemic—1 January 2015 to 10 March 2020; (2) during the COVID-19 pandemic—11 March 2020 to 4 May 2023; and (3) after the COVID-19 pandemic—5 May 2023 to 31 December 2024.

### 2.5. Data Management Plan

Before entering into the database, an enrollment code was generated for all neonates included in the study. The questionnaire used in the study had four sections. The first part of the questionnaire was used to enter patient data, which was collected from the patient’s medical records. The second part collected data regarding the neonate’s vaccination status. We checked whether the neonate was vaccinated with the BCG and hepatitis B vaccines. If not, the reason was determined (lack of parental consent, not qualified due to medical consent). The third part of the questionnaire assessed the patient’s suitability for the study (checking for missing data). In the fourth part, the patient was assigned to the appropriate group (A–C), and then to a subgroup (B1–B3).

### 2.6. Sample Size and Missing Data Points

Because of the nature of the study, sample size calculations were not necessary. All available data from the medical records were used. To handle missing data, we used a “complete case analysis” and excluded all cases with missing values. Full data was not available in the case of 5 neonates. Therefore, we excluded them from the analysis (0.02% of all neonates primary included in the study). To avoid overfitting in logistic regression, we used the 10 Events Per Variable (EPV) rule to establish an adequate number of events per independent variable [13].

### 2.7. Data Quality Control

We trained the data collectors and supervisors for 3 days. The procedures of data collection, handling, and storage were discussed in detail. The principal investigator and supervisors monitored the process of data collection. When the questionnaires were completed, they were checked for data completeness and consistency. Correction of missing data was made before data analysis.

### 2.8. Operational Definitions

The neonates were classified based on their gestational age (Table 1.)

City population: small city—fewer than 20,000 inhabitants, medium-sized city—at least 20,000 but fewer than 100,000 inhabitants, and big city—at least 100,000 inhabitants.

### 2.9. Statistical Analysis

Initially, we performed descriptive analyses. Categorical variables were expressed using frequency and percentage (%). To check if the data were normally distributed, we used the Shapiro–Wilk test. As the data did not follow a normal distribution, quantitative variables were presented as median and interquartile range (Q1–Q3).

To analyze categorical data and determine if two categorical variables were associated we used the Chi-square test or the Fisher’s exact test (for small sample sizes). Since the data did not follow a normal distribution, the U Mann–Whitney test was used to compare continuous and ordinal variables between two independent groups. Additionally, simplified rank-biserial coefficient of correlation based on the U statistic was used as a non-parametric measure of effect size to indicate the strength and the direction of the relationship between a binary variable and a continuous or ordinal variable.

Then uni- and multivariate logistic regression analysis were performed to assess the relationship between immunization status (dependent variable) and neonate’s characteristic (predictors: (1) mother’s age, (2) place of residence, (3) gravidity, (4) parity, (5) type of pregnancy, (6) way of delivery, (7) gestational age, (8) sex). Separate analyses were performed for neonates not fully vaccinated due to parents’ decision. Therefore, the following three subgroups were analyzed: B1—neonates, who did not receive the BCG and hepatitis B vaccines (N = 720), B2—neonates, who did not receive the BCG vaccine (N = 108), B3—neonates, who did not receive the hepatitis B vaccine (N = 137). To avoid overfitting and establish an adequate number of events per independent variable we used the 10 Events Per Variable (EPV) rule. In the logistic regression analysis, 8 predictors were analyzed. Therefore, the minimum number of observations in the least frequent outcome category was 80. In our study, the smallest group analyzed was the subgroup B2 (N = 108), which met the criteria of the 10 EPV rule. To eliminate confounding, we included only independent variables with *p*-values <0.20 in multivariate logistic regression analysis. Unadjusted odds ratios (COR), adjusted odds ratios (AOR), 95% confidence intervals (CI), and *p*-values at AOR were calculated. The backward stepwise model was applied in multivariate logistic regression model.

All statistical tests were two-tailed. We used an alpha of 0.05 and when *p*-value was <0.05 the differences were considered statistically significant. Statistical analysis was performed with the Statistical 13.3 Package (TIBCO Software Inc., San Ramon, CA, USA).

### 2.10. Ethical Considerations

The study protocol was approved by The Local Bioethics Committee of the Medical University of Bialystok (protocol code: APK.002.35.2024, date of approval: 18 January 2024). All procedures were conducted according to the Declaration of Helsinki. Patient informed consent to participate in this study was waived by The Local Bioethics Committee of the Medical University of Bialystok.

## 3. Results

### 3.1. The Characteristics of the Study Group

Between 1 January 2015 and 31 December 2024, 21,102 neonates were admitted to the Department. The number of neonates admitted in particular years was as follows: 2015–1821, 2016–1962, 2017–2214, 2018–2513, 2019–2287, 2020–2141, 2021–2084, 2022–2072, 2023–2072, 2024–1936. Accordingly, 88.35% of the neonates (N = 18,643) were vaccinated with both the BCG and hepatitis B vaccines (group A), 39.24% of the remaining neonates (N = 965) were not fully vaccinated due to parents’ decision (group B), and 60.75% (N = 14,949) were not qualified for vaccination (group C). The following percentages of all neonates received the BCG and hepatitis B vaccines: 89.00% (N = 18,780) and 88.86% (18,751), respectively. The characteristics of these three groups as well as their comparison with all neonates are presented in Table 2.

The neonates with the following characteristics were more likely to not be vaccinated due to parents’ decision: term neonates (*p* = 0.029) born to multigravida (*p* = 0.000) and multiparous (*p* = 0.000) mothers aged ≥35 years (*p* = 0.000) and living in cities (*p* = 0.000). Contrary, preterm (*p* = 0.000), male (*p* = 0.00) neonates born from multiple pregnancies (*p* = 0.014) to mothers living in rural areas (*p* = 0.025) were more often not qualified for vaccination.

The groups A, B and C were then compared with each other. When contrasted with the group A, the group B was characterized by a higher percentage of neonates born to multigravida (*p* = 0.000) and to multiparous (*p* = 0.000) mothers aged ≥35 years (*p* = 0.000) and living in a city (*p* = 0.000). Neonates from the group B were also characterized by a higher maternal age (*p* = 0.000). No differences were observed for other variables. When compared to the group A, the group C was characterized by a higher percentage of preterm (*p* = 0.000) and male (*p* = 0.000) neonates born to mothers with multiple pregnancies (*p* = 0.007) and to mothers aged ≥35 years (*p* = 0.044). Neonates from the group C were also characterized by a lower 1 min Apgar score (*p* = 0.000), gestational age (*p* = 0.000), and birth weight (*p* = 0.000). No differences were observed for other variables.

### 3.2. The Characteristics of the Neonates Unvaccinated Due to Parents’ Decision

Neonates who did not receive both the BCG and hepatitis B vaccines accounted for 74.61% (N = 720), 11.19% of the neonates (N = 108) did not receive the BCG vaccine, and 14.20% (N = 137) did not receive the hepatitis B vaccine. The characteristics of these three groups as well as their comparison with all neonates are presented in Table 3.

The neonates with the following characteristics were more likely to not receive both vaccines: term neonates (*p* = 0.002) born to multigravida (*p* = 0.000) and multiparous (*p* = 0.000) mothers aged ≥35 years (*p* = 0.000) and living in cities (*p* = 0.020). Preterm (*p* = 0.000) neonates born from multiple pregnancies (*p* = 0.020) to multigravida (*p* = 0.000) and multiparous (*p* = 0.002) mothers living in cities (*p* = 0.048) more often did not receive the BCG vaccine. In the case of the hepatitis B vaccine, term neonates (*p* = 0.025) born to mothers living in cities (*p* = 0.015) were more likely to not receive this vaccine. The subgroups were then compared with each other.

When contrasted to the subgroup B1, the subgroup B2 was characterized by a higher percentage of preterm neonates (*p* = 0.000). The neonates from the subgroup B2 were also characterized by lower birth weight (*p* = 0.003), gestational age (*p* = 0.029), and 1 min Apgar score (*p* = 0.001). No differences were observed for other variables. When compared to the subgroup B1, the subgroup B3 was characterized by a lower percentage of neonates born to primigravida (*p* = 0.003) and primiparous (*p* = 0.001) mothers as well as a higher 1 min Apgar score (*p* = 0.029). No differences were observed for other variables. When compared to the subgroup B2, the subgroup B3 was characterized by a higher percentage of term neonates (*p* = 0.000) born from single pregnancies (*p* = 0.032) to primigravida (*p* = 0.003) and primiparous (*p* = 0.005) mothers. The neonates from the subgroup B3 were also older (*p* = 0.017) and received higher 1 min Apgar score (*p* = 0.000).

### 3.3. Factors Associated with the Immunization Status—The Multivariate Logistic Regression Analysis

In the final model of multivariate logistic regression analysis, the following variables were associated with the immunization status ‘did not receive the BCG and hepatitis B vaccines’: mother’s age (*p* = 0.004), place of residence (*p* = 0.012), parity (*p* = 0.002), and gestational age (*p* = 0.000). Term neonates born to multiparous mothers aged ≥35 years and living in cities were more likely to not receive both vaccines. The immunization status ‘did not receive the BCG vaccine’ was associated with place of residence (*p* = 0.049), gravidity (*p* = 0.000), and gestational age (0.003). Preterm neonates born to multiparous mothers living in cities were more likely to not receive the BCG vaccine. In the case of neonates who ‘did not receive the hepatitis B vaccine’, the following variables were associated with their immunization status: place of residence (*p* = 0.019) and gestational age (*p* = 0.034). Term neonates born to mothers living in cities were more likely to not receive the hepatitis B vaccine. Detailed data are presented in Table 4.

### 3.4. The Analysis of the Immunization Status of the Neonates in Individual Years

**Figure 2:**
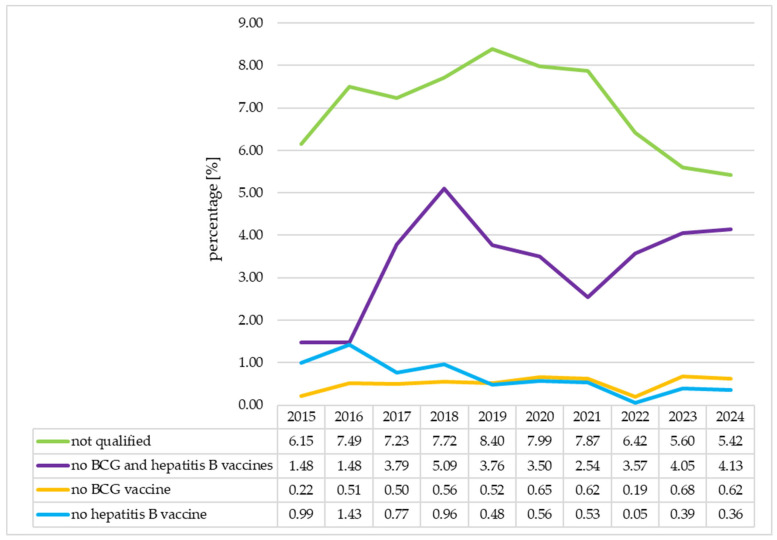
The percentages of neonates not qualified for vaccination and not vaccinated in individual years. BCG—Bacillus Calmette-Guérin.

The percentages of neonates not qualified for vaccination and not vaccinated with both vaccines, the BCG vaccine and the hepatitis B vaccine were analyzed in individual years. The percentage of neonates not qualified for vaccination increased between 2015 and 2019 (6.15% vs. 5.43%, *p* = 0.006). Then, it decreased between 2019 and 2024 (8.40% vs. 5.43%, *p* = 0.000). There was a significant increase in the percentage of neonates not vaccinated with both vaccines between 2015 and 2018 (1.45% vs. 5.09%, *p* = 0.000). Then, between 2018 and 2021, this percentage decreased significantly (5.09% vs. 2.54%, *p* = 0.000) and increased again between 2021 and 2024 (2.54% vs. 4.13%, *p* = 0.005). The percentage of neonates who did not receive the BCG vaccine increased between 2015 and 2024 (0.22% vs. 0.62%, *p* = 0.036). However, in 2022, there was a significant decline in the percentage of neonates from this group (2021 vs. 2022: 0.62% vs. 0.19%, *p* = 0.049), which returned, in the following year, to values similar to those from before 2022 (2022 vs. 2023: 0.19% vs. 0.68%, *p* = 0.031). The percentage of neonates who did not receive the hepatitis B vaccine decreased between 2015 and 2024 (0.99% vs. 0.36%, *p* = 0.025). In this case, there was a similar significant decline in 2022 and an increase the following year (2021 vs. 2022: 0.53% vs. 0.05%, *p* = 0.006; 2022 vs. 2023: 0.05% vs. 0.39%, *p* = 0.039). Detailed data are presented in Figure 2.

### 3.5. The Analysis of the Immunization Status of the Neonates According to Their Gestational Age

**Figure 3:**
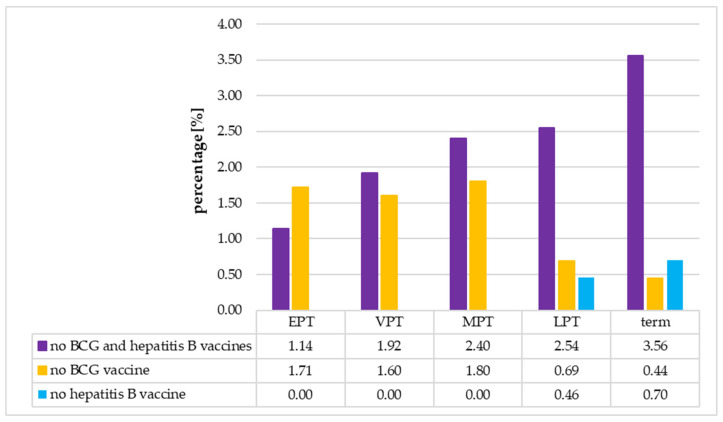
The percentage of non-vaccinated neonates in individual age groups. BCG—Bacillus Calmette-Guérin, EPT—extremely preterm, VPT—very preterm, MPT—moderate preterm, LPT—late preterm.

The percentage of neonates not vaccinated with both vaccines increased with the increase in gestational age of the neonates; however, the difference between EPT and term neonates was not significant (EPT vs. term neonates: 1.14% vs. 3.56%, *p* = 0.097 (Fisher’s exact test)). Contrary to this, the percentage of neonates not vaccinated with the BCG vaccine decreased significantly with the increase in gestational age of the neonates (EPT vs. term neonates: 1.71% vs. 0.44%, *p* = 0.045 (Fisher’s exact test)). In the group of neonates not vaccinated with the hepatitis B vaccine, there were only LPT and term neonates. Detailed data are presented in Figure 3.

### 3.6. The Analysis of the Immunization Status of the Neonates According to Their Place of Residence

**Figure 4:**
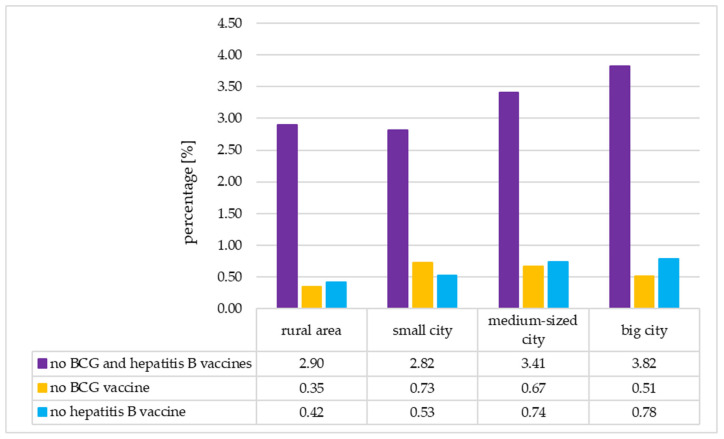
Percentage of unvaccinated newborns by place of residence. BCG—Bacillus Calmette-Guérin.

The percentage of neonates not vaccinated with both vaccines was similar and the lowest in neonates of mothers living in rural areas and small cities. Then, it increased with the increase in city population (rural area vs. big city: 2.90% vs. 3.82%, *p* = 0.003 (Chi-square test)). A similar trend was seen in the percentage of neonates not vaccinated with the hepatitis B vaccine (rural area vs. big city: 0.42% vs. 0.78%, *p* = 0.007 (Chi-square test)). Contrary, no differences related to the place of residence were observed for the percentage of neonates not vaccinated with the BCG vaccine (*p* < 0.050 in each case (Chi-square test)). Detailed data are presented in Figure 4.

### 3.7. The Analysis of the Neonatal Immunization Before, During and After the COVID-19 Pandemic

**Figure 5:**
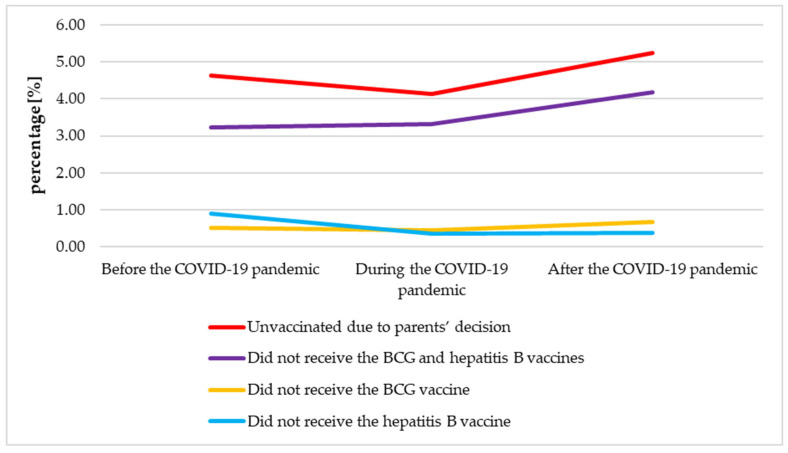
Neonatal immunization before, during and after the COVID-19 pandemic. BCG—Bacillus Calmette-Guérin; COVID-19—Coronavirus disease 2019.

The percentage of neonates unvaccinated due to parents’ decision was significantly higher in the post-pandemic period than during the pandemic (*p* = 0.012). The percentage of neonates who did not receive the BCG and hepatitis B vaccines was significantly higher after the COVID-19 pandemic, when compared to the pre-pandemic period (*p* = 0.009). Contrary, when compared to the pre-pandemic period, the percentage of neonates who did not receive the hepatitis B vaccine was significantly lower during the pandemic and in the post-pandemic period (*p* = 0.000 and *p* = 0.003, respectively). The percentage of neonates not vaccinated with both vaccines did not change significantly in the periods studied. Detailed data are presented in Table 5. Time trends were presented graphically in Figure 5.

### 3.8. Summary of Findings

During the study period, 88.35% of the neonates received both the BCG and hepatitis B vaccines. 39.24% of the unvaccinated neonates were due to parental refusal and 74.61% of this subgroup did not receive both vaccines.

Term neonates born to multiparous mothers aged ≥35 years and living in cities were more likely to not receive both vaccines (*p* < 0.05). Additionally, preterm neonates born to multiparous mothers living in cities were more likely to not receive the BCG vaccine (*p* < 0.05) and term neonates born to mothers living in cities were more likely to not receive the hepatitis B vaccine (*p* < 0.05).

The percentage of neonates not vaccinated with both vaccines increased with the rise in city population (*p* < 0.05) and in gestational age. However, the second relationship was not statistically significant (*p* < 0.05).

Between 2015 and 2024, the percentage of neonates who did not receive the BCG vaccine increased and the percentage of neonates who did not receive the hepatitis B vaccine decreased (*p* < 0.05). The percentage of neonates not vaccinated with both vaccines, after a significant drop between 2018 and 2021, exhibited significant growth between 2021 and 2024 (*p* < 0.05).

The percentage of neonates who did not receive the BCG and hepatitis B vaccines was considerably higher after the COVID-19 pandemic, when compared to the pre-pandemic period (*p* < 0.05). Contrary, when compared to the pre-pandemic period, the percentage of neonates who did not receive the hepatitis B vaccine was significantly lower during the pandemic and in the post-pandemic period (*p* < 0.05). The percentage of neonates not vaccinated with both vaccines did not change notably in the periods studied.

## 4. Discussion

We attempted to look at the issue of neonatal vaccinations from a different perspective. There are numerous studies in the literature examining parents’ willingness to vaccinate their neonates, but data on neonates already vaccinated is scarce. Therefore, we wanted to present the problem of vaccine hesitancy from the perspective of a vaccinated or unvaccinated neonate, to highlight aspects that may influence the final stage of a decision-making process: the vaccination itself.

In our study, we assessed 21,102 neonates admitted to the Department of Neonatology and Neonatal Intensive Care of the Medical University of Bialystok between 2015 and 2024. During the study period, 90.00% of the neonates received the BCG vaccine and 88.86% received the hepatitis B vaccine. According to the data from the Central Statistical Office in Poland, between 2015 and 2023, 91.60% of children were vaccinated with the BCG vaccine and 97.10% with the primary hepatitis B vaccine. Also, there was a downward trend in the percentages of BCG- and hepatitis B-vaccinated children in the total number of births (the BCG vaccine: from 92.60% in 2015 to 90.90% in 2023; the hepatitis B vaccine: from 98.80% in 2015 to 97.40% in 2023) [14]. These percentages were substantially higher than in our study, most likely due to differences in methodology. We assessed the immunization status of neonates in the Department, shortly after birth, while the Central Statistical Office examined vaccination coverage with the BCG vaccine in the first year of life and with the hepatitis B vaccine in the first two years of life.

Using the logistic regression, we tried to answer the following question: what characteristics are associated with the lack of parental consent to vaccinate a neonate. We found that the following variables were associated with the immunization status ‘did not receive the BCG and hepatitis B vaccines’ due to parents decision: gestational age (*p* = 0.000), parity (*p* = 0.002), mother’s age (*p* = 0.004), and place of residence (*p* = 0.012). Term neonates born to multiparous mothers aged ≥35 years and living in cities were more likely to not receive both the BCG and hepatitis B vaccines. At this point, it is important to highlight that vaccination attitudes differ significantly depending on the characteristics of the local population [15]. This makes it difficult to generalize the results we present and to compare them with the results of other researchers.

Firstly, term neonates are more likely to not receive both vaccines. However, there is very little data on this issue in the literature. Van de Berg et al. assessed parents’ attitudes toward the COVID-19 vaccine in Munich, Germany. He found that a child belonging to a clinically vulnerable group may be a positive determinant of vaccination [16]. This relationship can be applied to the situation observed in our study. When a neonate’s life is at risk due to prematurity, a change in parental attitudes toward vaccinations is evident. When parents see that their child is so young and has breathing, circulatory, and feeding problems, their fear of vaccinations seems to be replaced by the real threat of not vaccinating their child.

Secondly, neonates born to multiparous mothers are more likely to not receive both vaccines. Numerous studies have shown that confidence in vaccinations is higher in families with only one child [17,18,19,20,21]. This may be attributed to experiences with vaccine-preventable diseases or to a vaccination itself (side effects) in previous children. Moreover, primiparous and multiparous women make decisions regarding vaccination of neonates differently. Corben et al. showed that experienced mothers were more likely to make the decision about vaccination before or during pregnancy (94.5%), while first-time mothers were less likely to do so (77.5%) and experienced more decision-making conflicts. Therefore, it seems more possible for a healthcare professional to educate and convince a first-time mother about the safety and effectiveness of vaccination as well as convince her to vaccinate a neonate [22].

Thirdly, neonates of mothers aged ≥35 are more likely to not receive both vaccines. Age differences in vaccine hesitancy were shown in an Australian study by Sharif-Nia et al. in which younger parents were less vaccine-hesitant than parents between 40 and 60 years. This phenomenon may also be explained by experiences with vaccine-preventable diseases or to the vaccination itself as younger parents are less experienced with infectious diseases and therefore feel more afraid of them [23]. A similar trend was found by Thirunavukkarasu et al. who analyzed maternal perception, hesitancy and satisfaction toward childhood immunization in Eastern Saudi Arabia. They found that vaccine hesitancy was associated with maternal age and, when compared to mothers aged <25 years, it was significantly higher in mothers aged 25–35 years (*p* = 0.003) and >35 years (*p* = 0.034) [24]. Similar conclusions were made by Soysal et al. investigating vaccine hesitancy and refusal among parents of children under five [25]. Moreover, in a Polish review by Szalast et al. assessing parental attitudes toward children’s vaccination, parents that were older were much more likely to not vaccinate their child [4]. However, study results are inconsistent and show varying associations between maternal age and vaccine hesitancy. This may be due to cultural differences between study locations [26,27].

Fourthly, neonates of mothers living in cities are more likely not to receive both vaccines. A study by Łoś-Rycharska et al. that analyzed parental knowledge of anti-vaccination movements in Poland showed that anti-vaccination arguments are significantly more common among parents living in big cities than small towns and rural areas (*p* < 0.001) [17]. This may translate into more frequent lack of parental consent to vaccination in children from cities. Moreover, individuals more engaged in social media tend to be more vaccine-hesitant as they may be confused by fake news and misinformation [23]. According to Gołębiewska et al., the use of social media is more common among people living in cities, which may additionally cause greater reluctance to vaccinations in this group [28]. However, in a polish study of Nylen et al. analyzing attitudes of new mothers toward childhood vaccinations in Rzeszów, Poland, demographic data analysis revealed no significant association between place of residence and willingness to vaccinate a child [29].

Additionally, we analyzed how the number of neonates not vaccinated with both vaccines changed over the years. We paid special attention to the COVID-19 pandemic. During the first year of the COVID-19 pandemic (2020), a significant decrease in parental vaccine hesitancy was observed. However, contrary to our findings, many countries reported a decline in routine vaccination coverage during the first year of the COVID-19 pandemic [30]. In the case of our population of neonates, this may be partly explained by reports of a beneficial effect of the BCG vaccine on the course of COVID-19 infection. However, due to the limited number of clinical studies and the lack of clear evidence, this effect appears to be nonspecific and remains controversial [31,32,33]. This phenomenon could also be caused by fear of an infectious disease spreading out of control. In this case, parents’ concerns about vaccination complications were overshadowed by the real threat of the COVID-19 virus. Nevertheless, since the beginning of 2021, there has been a steady increase in the number of neonates unvaccinated with both vaccines. This has likely resulted from declining trust in the implementation of mandatory vaccinations probably caused by a series of misinformation regarding COVID-19 vaccinations that accompanied the pandemic [34]. It appears that this may have translated into concerns about BCG and hepatitis B vaccinations.

Parental vaccine hesitancy is an important and growing problem; therefore, novel strategies are needed to counteract this issue. Nowadays, it is proposed to use tailored materials targeted at specific groups. For this reason, it is extremely important to conduct similar analyses in various areas in order to be able to properly adjust health policy programs aimed at vaccinations [24,35,36]. Knowledge about vaccinations should be easily accessible to parents, especially on the hospital’s social media, where they can obtain reliable information on this subject [37]. The percentage of unvaccinated children varies depending on the mother’s age and parity [17]. Therefore, we believe it is particularly important to reach more experienced mothers who already have children. We believe it would be valuable to continually improve parental education by emphasizing that each neonate has a different immune response and may react differently to infectious diseases, and that this response does not necessarily reflect the response observed in their siblings. By implementing these measures, we aim to reduce concerns about vaccinations, which may stem from parents’ experiences with the disease and the vaccine itself. The percentage of unvaccinated children also varies depending on the child’s health [16]. We believe that it is equally relevant to explain that every neonate, regardless of their current health status, should have the opportunity to have their health protected. Of particular concern is the higher proportion of full-term neonates who remain unvaccinated, despite clear eligibility for routine immunization.

## 5. Strengths and Limitations

Our assessment of neonates from the main tertiary referral hospital in north-eastern Poland, a representative of medical facilities offering neonatal care in this area, allows for a detailed insight into their needs and complements other data, such as mortality analyses, which are essential for the implementation of effective health policy programs [35]. In our study, we reviewed all neonates which were admitted to the Department from 2015 to 2024 and therefore eliminated potential sampling error. However, this study has several limitations which should be mentioned. Our results should not be generalized to the wider neonatal population. We analyzed the trends and determinants of neonatal vaccination in a major tertiary referral hospital in north-east Poland; therefore, our results were probably influenced by the specificity of the center where the research was conducted as well as by the characteristics of the community living in a given area. In addition, we focused on two vaccinations that are administered in the Department and did not follow up on the neonate after discharge or transfer to another hospital/ward. Thus, we cannot determine whether the neonates were vaccinated at a later date and our results may not accurately reflect the vaccination coverage of neonates. It is likely that some neonates who did not receive vaccines in our Department will be vaccinated later. Therefore, the actual percentage of unvaccinated neonates may be lower than that reported in our study. We based our study on retrospective data and, despite all efforts, we cannot rule out potential errors related to data collection and missing data in the documentation. Finally, our study was limited to the data documented in the medical records, so other variables that could have influenced the parent’s decision may have been omitted.

## 6. Conclusions

The analysis revealed a specific group of neonates who are at risk of not receiving both vaccines: term neonates born to multiparous mothers aged ≥35 years and living in cities. These results may suggest which patients should be particularly taken into account when designing strategies to increase vaccine acceptance in the area covered by the study. However, it should be emphasized that due to differences in communities, similar studies should be carried out in different parts of the world.

## Figures and Tables

**Table 1:** Classification of neonates at birth based on gestational age.

Terminology	Abbreviation	Gestational Age (Weeks)
term	-	37 0/7–41 6/7
late preterm (LPT)	LPT	34 0/7–36 6/7
moderate preterm	MPT	32 0/7–33 6/7
very preterm	VPT	28 0/7–31 6/7
extremely preterm	EPT	≤27 6/7

LPT—late preterm, MPT—moderate preterm, VPT—very preterm, EPT—extremely preterm.

**Table 2:** The characteristics of the study group.

Characteristic	All Neonates (N = 21,102)	Group A: Vaccinated (N = 18,643)	Unvaccinated (N = 2459)		*p*		
			Group B: Parents’ Decision (N = 965)	Group C: Not Qualified (N = 1494)			
	Frequency (Percentage of all Neonates, %)				p1	p2	p3
male	10,750 (50.94)	9358 (44.35)	500 (2.37)	892 (4.23)	0.133	0.600	0.000
female	10,349 (49.04)	9285 (44.00)	465 (2.20)	599 (2.84)			
vaginal delivery	11,094 (52.57)	9811 (46.49)	509 (2.41)	774 (3.67)	0.917	0.834	0.567
cesarean delivery	10,008 (47.43)	8832 (41.85)	456 (2.16)	720 (3.41)			
single pregnancy	19,443 (92.14)	17,211 (81.56)	882 (4.18)	1350 (6.40)	0.502	0.405	0.014
multiple pregnancy	1659 (7.86)	1432 (6.79)	83 (0.39)	144 (0.68)			
place of residence: city	15,627 (74.05)	13,796 (65.38)	764 (3.62)	1067 (5.06)	0.903	0.000	0.025
place of residence: rural area	5475 (25.95)	4847 (22.97)	201 (0.95)	427 (2.02)			
primigravida	8222 (38.96)	7356 (34.86)	277 (1.31)	589 (2.79)	0.314	0.000	0.724
multigravida	12,880 (61.04)	11,287 (53.49)	688 (3.26)	905 (4.29)			
primiparous	9603 (45.51)	8573 (40.63)	335 (1.59)	695 (3.29)	0.340	0.000	0.448
multiparous	11,499 (54.49)	10,070 (47.72)	630 (2.99)	799 (3.79)			
term birth	18,553 (87.92)	16,712 (79.20)	871 (4.13)	970 (4.60)	0.000	0.029	0.000
preterm birth	2549 (12.08)	1931 (9.15)	94 (0.45)	524 (2.48)			
maternal age: <35 years	15,814 (74.94)	14,051 (66.59)	672 (3.18)	1091 (5.17)	0.324	0.000	0.099
maternal age: ≥35 years	5288 (25.06)	4592 (21.76)	293 (1.39)	403 (1.91)			
	**Median (Q1–Q3)**				**p1**	**p2**	**p3**
birth weight (g)	3370 (2980 −3710)	3400 (3000–3730)	3360 (3000–3700)	3120 (2200–3650)	0.004 (0.017)	0.963 (0.001)	0.000 (0.208)
gestational age (weeks)	39.00 (38.00–40.00)	39 (38–40)	39 (38–40)	38 (35–40)	0.001 (0.019)	0.274 (0.021)	0.000 (0.250)
1 min Apgar score	10.00 (9.00–10.00)	10 (9–10)	10 (9–10)	9 (6–10)	0.000 (0.026)	0.334 (0.018)	0.000 (0.336)

p1—comparison of all neonates and the group A; p2—comparison of all neonates and the group B; p3—comparison of all neonates and the group C; Q1–Q3—interquartile range; r—simplified rank-biserial coefficient of correlation based on the U statistic.

**Table 3:** The characteristics of the neonates unvaccinated due to parents’ decision.

Characteristic	All Neonates (N = 21,102)	Unvaccinated—Parents’ Decision (N = 965)			*p*		
		Subgroup B1: Did not Receive the BCG and Hepatitis B Vaccines (N = 720)	Subgroup B2: Did not Receive the BCG Vaccine (N = 108)	Subgroup B3: Did not Receive the Hepatitis B Vaccine (N = 137)			
	Frequency (Percentage of all Neonates, %)				p1	p2	p3
male	10,750 (50.94)	371 (1.76)	59 (0.28)	70 (0.33)	0.761	0.446	0.973
female	10,349 (49.04)	349 (1.64)	49 (0.23)	67 (0.32)			
vaginal delivery	11,094 (52.57)	384 (1.82)	56 (0.27)	69 (0.33)	0.689	0.881	0.606
cesarean delivery	10,008 (47.43)	336 (1.59)	52 (0.25)	68 (0.32)			
single pregnancy	19,443 (92.14)	660 (3.13)	93 (0.44)	129 (0.61)	0.644	0.020	0.380
multiple pregnancy	1659 (7.86)	60 (0.28)	15 (0.07)	8 (0.04)			
place of residence: city	15,627 (74.05)	561 (2.66)	89 (0.42)	114 (0.54)	0.020	0.048	0.015
place of residence: rural area	5475 (25.95)	159 (0.75)	19 (0.09)	23 (0.11)			
primigravida	8222 (38.96)	198 (0.94)	24 (0.11)	55 (0.26)	0.000	0.000	0.777
multigravida	12,880 (61.04)	522 (2.47)	84 (0.40)	82 (0.39)			
primiparous	9603 (45.51)	236 (1.12)	33 (0.16)	66 (0.31)	0.000	0.002	0.532
multiparous	11,499 (54.49)	484 (2.29)	75 (0.36)	71 (0.34)			
term birth	18,553 (87.92)	660 (3.13)	82 (0.39)	129 (0.61)	0.002	0.000	0.025
preterm birth	2549 (12.08)	60 (0.28)	26 (0.12)	8 (0.04)			
maternal age: <35 years	15,814 (74.94)	495 (2.35)	75 (0.36)	102 (0.48)	0.000	0.189	0.869
maternal age: ≥35 years	5288 (25.06)	225 (1.07)	33 (0.16)	35 (0.17)			
	**Median (Q1–Q3)**				**p1 (r1)**	**p2 (r2)**	**p3 (r3)**
birth weight (g)	3370 (2980 −3710)	3400 (3050–3700)	3175 (2610–3700)	3300 (2950–3700)	0.195 (0.028)	0.007 (0.148)	0.596 (0.026)
gestational age (weeks)	39.00 (38.00–40.00)	39 (38–40)	39 (37–40)	39 (38–40)	0.205 (0.028)	0.072 (0.100)	0.109 (0.079)
1 min Apgar score	10.00 (9.00–10.00)	10 (9–10)	9 (8–10)	10 (9–10)	0.291 (0.023)	0.008 (0.151)	0.010 (0.127)

BCG—Bacillus Calmette-Guérin; p1—comparison of all neonates and the subgroup B1; p2—comparison of all neonates and the subgroup B2; p3—comparison of all neonates and the subgroup B3; Q1–Q3—interquartile range; r—simplified rank-biserial coefficient of correlation based on the U statistic.

**Table 4:** The relationship between the immunization status and the neonate’s characteristic.

Variables	Categories	Univariate Logistic Regression		Multivariate Logistic Regression (Backward Stepwise Model)	
		COR (CI = 95%)	*p*-Value	AOR (CI = 95%)	*p*-Value
Immunization status: “did not receive the BCG and hepatitis B vaccines” (N = 720)					
mother’s age	≥35 years	1.439 (1.224–1.691)	0.000	1.280 (1.084–1.511)	0.004
	<35 years	1		1	
place of residence	city	1.245 (1.041–1.489)	0.016	1.260 (1.053–1.507)	0.012
	rural area	1		1	
gravidity	multigravida	1.712 (1.450–2.021)	0.000	-	-
	primigravida	1			
parity	multiparous	1.733 (1.480–2.030)	0.000	1.524 (1.053–2.029)	0.002
	primiparous	1		1	
type of pregnancy	multiple	1.069 (0.817–1.399)	0.626	-	-
	single	1			
way of delivery	cesarean section	0.968 (0.834–1.124)	0.671	-	-
	vaginal	1			
gestational age	term	1.529 (1.170–1.998)	0.002	1.658 (1.410–1.950)	0.000
	preterm	1		1	
sex	male	1.024 (0.883–1.189)	0.751	-	-
	female	1			
Immunization status: “did not receive the BCG vaccine” (N = 108)					
mother’s age	≥35 years	1.413 (0.937–2.130)	0.099	-	-
	<35 years	1			
place of residence	city	1.645 (1.002–2.702)	0.049	1.749 (1.064–2.875)	0.028
	rural area	1		1	
gravidity	multigravida	2.242 (1.423–3.533)	0.001	2.265 (1.437–3.569)	0.000
	primigravida	1		1	
parity	multiparous	1.904 (1.263–2.871)	0.002	-	-
	primiparous	1			
type of pregnancy	multiple	1.901 (1.099–3.287)	0.022	-	-
	single	1			
way of delivery	cesarean section	1.194 (0.818–1.743)	0.358	-	**-**
	vaginal	1			
gestational age	term	0.430 (0.276–0.670)	0.000	0.471 (0.291–0.763)	0.003
	preterm	1		1	
sex	male	0.443 (0.794–1.696)	0.443	-	-
	female	1			
Immunization status: “did not receive the hepatitis B vaccine” (N = 137)					
mother’s age	≥35 years	1.058 (0.717–1.561)	0.775	-	-
	<35 years	1			
place of residence	city	1.742 (1.112–2.730)	0.015	1.714 (1.094–2.686)	0.019
	rural area	1		1	
gravidity	multigravida	0.951 (0.675–1.340)	0.776	-	-
	primigravida	1			
parity	multiparous	0.898 (0.642–1.257)	0.531	-	-
	primiparous	1			
type of pregnancy	multiple	0.726 (0.355–1.486)	0.381	-	-
	single	1			
way of delivery	cesarean section	1.125 (0.804–1.574)	0.492	-	-
	vaginal	1			
gestational age	term	2.222 (1.087–4.544)	0.029	2.170 (1.061–4.438)	0.034
	preterm	1		1	
sex	male	1.006 (0.719–1.408)	0.972	-	-

BCG—Bacillus Calmette-Guérin; COR—crude odds ratio; AOR—adjusted odds ratio; CI—confidence interval.

**Table 5:** Neonatal immunization before, during and after the COVID-19 pandemic.

Group of Neonates	Before the COVID-19 Pandemic (N = 11265)	During the COVID-19 Pandemic (N = 6591)	After the COVID-19 Pandemic (N = 3246)	*p*		
	Frequency (Percentage of all Neonates, %)			p1	p2	p3
Group A: Vaccinated	9908 (87.95)	5827 (88.41)	2908 (89.59)	0.365	0.081	0.011
Group B: Unvaccinated due to parents’ decision	523 (4.64)	272 (4.13)	170 (5.24)	0.107	0.012	0.162
Subgroup B1: Did not receive the BCG and hepatitis B vaccines	365 (3.24)	219 (3.32)	136 (4.19)	0.765	0.030	0.009
Subgroup B2: Did not receive the BCG vaccine	57 (0.51)	29 (0.44)	22 (0.68)	0.539	0.123	0.241
Subgroup B3: Did not receive the hepatitis B vaccine	101 (0.90)	24 (0.36)	12 (0.37)	0.000	0.966	0.003
Group C: Not qualified for vaccination	834 (7.40)	492 (7.46)	168 (5.18)	0.880	0.000	0.000

BCG—Bacillus Calmette-Guérin; COVID-19—Coronavirus disease 2019; p1—comparison of variables before and during the COVID-19 pandemic; p2—comparison of variables during and after the COVID-19 pandemic; p3—comparison of variables before and after the COVID-19 pandemic.

## Data Availability

The data that support the findings of this study are not openly available due to reasons of sensitivity and are available from the corresponding author upon reasonable request.
